# The prognostic value of leucine-rich repeat-containing G-protein (Lgr5) and its impact on clinicopathological features of colorectal cancer

**DOI:** 10.1007/s00432-020-03314-7

**Published:** 2020-07-15

**Authors:** Arkadiusz Gzil, Izabela Zarębska, Damian Jaworski, Paulina Antosik, Justyna Durślewicz, Joanna Maciejewska, Ewa Domanowska, Natalia Skoczylas-Makowska, Navid Ahmadi, Dariusz Grzanka, Łukasz Szylberg

**Affiliations:** 1 Department of Clinical Pathomorphology, Collegium Medicum in Bydgoszcz, Sklodowskiej-Curie Str. 9, 85-094 Bydgoszcz, Poland; 2 Nicolaus Copernicus University, Toruń, Poland; 3 Department of Pathomorphology, Military Clinical Hospital, Bydgoszcz, Poland; 4 Chair and Department of Oncologic Pathology and Prophylactics, Greater Poland Cancer Center, Poznan University of Medical Sciences, Poznan, Poland; 5 Department of Tumor Pathology and Pathomorphology, Oncology Center, Prof. Franciszek Łukaszczyk Memorial Hospital, Bydgoszcz, Poland

**Keywords:** Lgr5, DCLK1, ANAX2, CD44, CSC, Colorectal cancer

## Abstract

**Introduction:**

Colorectal cancer (CRC) constitutes one of the most prevalent malignancies in the world. Recent research suggests that cancer stem cells (CSCs) are responsible for tumor cell’s malignant behavior in CRC. This study has been designed to determinate clinical implications of CSC markers: CD44, DCLK1, Lgr5, and ANXA2 in CRC.

**Materials and methods:**

The study was performed on tissue samples which were collected from 89 patients undergoing colectomy. Formalin-fixed paraffin-embedded tissue blocks with representative tumor areas were identified and corded. Immunohistochemical staining was performed using anti-CD44, anti-LGR5, anti-ANXA2, and anti-DCLK1 antibodies. The H-score system was utilized to determine the immunointensity of CRC cells.

**Results:**

The lower expression of Lgr5 was significantly correlated with the presence of lymph-node metastases (*p* = 0.011), while high expression of Lgr5 was statistically significant in vascular invasion in examined cancer tissue samples (*p* = 0.027). Moreover, a high H-score value of Lgr5 expression was significantly related to a reduced overall survival rate (*p* = 0.043).

**Conclusion:**

Our results suggest a strong relationship between CSC marker Lgr5 and vascular invasion, presence of lymph-node metastasis, and overall poor survival. The presence of Lgr5 might be an unfavorable prognostic factor, and its high level in cancer tissue is related to an aggressive course. This marker could also be used to access the effectiveness of the treatment.

## Introduction

Colorectal cancer (CRC) constitutes one of the most prevalent malignancies in the world, contributing 9% of the total number of new cases diagnosed in 2018 in the United States (Siegel et al. 2019). Factors associated with an increased risk or development of CRC include obesity, physical inactivity, smoking, alcohol use, age, type 2 diabetes mellitus, and a family history of colon or rectal cancer (Marley and Nan 2016). It should be noted that CRC is a multifactorial disease, which is also connected with several genetic mutations such as changes in the APC, STK11, MYH, and mismatch repair genes (Genetics 2005). The staging system most often used for CRC is a classification system developed by the American Joint Committee on Cancer, which is based on three basic elements, first, the size and extent of the tumor (T), second, the number of nearby lymph nodes with cancer metastases (N), and, finally, distant metastases, for example, the spread of the primary tumor to other parts of the body (M). The 5-year survival rate for those diagnosed with early stage, localized disease (stages I and II) is approximately 90%; however, only about 39% of patients are diagnosed at this stage (Amin et al. 2017). Late-stage diagnosis (stages III and IV) of CRC is associated with a dramatically worse prognosis; the 5-year survival years fluctuate at around 13.1% (Simon 2016). Distant metastasis and recurrence are the major cause of patients' death, with more than 50% of CRC-related mortality are due to metastatic spread to the liver (Zarour et al. 2017).

Recent research suggests that cancer stem cells (CSCs) are responsible for tumor development and a tumor cell’s malignant behavior as well as metastasis in CRC (Ricci-Vitiani et al. 2009). CSCs are a subpopulation of cancer cells that have the ability of self-renewal, the potential to generate differentiated cells of the tissue of origin (multipotency), and resistance to chemotherapy, and have high tumorigenicity (Pang et al. 2010). Furthermore, it is well known that CSCs are insensitive to the current drug regimens. CSCs in CRC are identified via a group of surface markers, such as CD44, CD133, ANXA2, CD24, DCLK1, Lgr5, ALDH1, Nanog, Oct-4, SOX-2, and EpCAM (Leng et al. 2018) (Gzil et al. 2019a, b). Some of these markers could have a potential clinical role in predicting pathological stage, therapy resistance, and cancer recurrence in patients with colorectal carcinoma.

The current study has been designed to determinate clinical implications related to the expression level of chosen CSC markers of CRC and their importance in tumor progression and prognosis, which is still not well understood. The level of CSC markers such as CD44, DCLK1, Lgr5, and ANXA2 were investigated in correlation with clinical data and histopathological parameters. Moreover, to define the prognostic value of these proteins, the expression levels of investigated markers were compared with patient survival rate.

## Materials and methods

### Materials

The study was performed on tissue samples which were collected from 89 patients undergoing colectomy due to adenocarcinoma. The samples were excluded from further analysis; if patients received neoadjuvant chemotherapy before primary resection, the performed surgery was re-operated or resection of the recurrent tumor, and if the quality of the sample collected was unacceptable. The medical records include gender, age, pathological characteristic of resected material, and clinical outcome.

The investigated group consists of 37 female and 52 males. Excluded patients were diagnosed with an inherited predisposing condition for CRC development, such as familial adenomatous polyposis, hereditary nonpolyposis colorectal cancer, MUTYH-associated polyposis, or other polyposis syndromes. At the time of diagnosis, 18 patients suffered from type 2 diabetes mellitus, 9 were diagnosed with chronic kidney disease, and 26 had cardiovascular disease. Colorectal cancer manifested by anemia in 20 patients, and intestinal obstruction in 25 patients and in 6 perforation. In the case of 16 patients, first symptoms were related with the presence of distal metastasis, while 22 patients were diagnosed during screening program. Most common localizations of tumors were rectum (36%) and sigmoid colon (27%). Histopathological records revealed 16 cases (18%) with the invasion of muscularis propria (pT2), 58 cases (65%) with the invasion of subserosa or surrounding tissues (pT3), and 15 cases (17%) with the presence on visceral peritoneum or attached to neighboring structures (pT4). Metastases to regional lymph nodes were detected in 46 cases, while 43 patients (48%) had metastases to the liver (pM1a). 34 cases of metastases were present at the time of diagnosis (synchronous metastases), while 9 of the patients developed metastases after primary surgical treatment (metachronous metastasis). Median follow-up in investigated group amounted 19.8 months. The detailed clinical data of our groups are summarized in Table 1.

**Table 1:** Clinicopathological characteristic

Feature	Groups	*N* (%)
Age	< 65 years	37 (42%)
	≥ 65 years	52 (58%)
Gender	Female	37 (42%)
	Male	52 (58%)
Localization of tumor	Caecum	8 (9%)
	Ascending colon	13 (15%)
	Transverse colon	8 (9%)
	Descending colon	4 (4%)
	Sigmoid colon	24 (27%)
	Rectum	32 (36%)
Grade	Well/moderated	82 (92%)
	poor	7 (8%)
T parameter	T2	16 (18%)
	T3	58 (65%)
	T4	15 (17%)
N parameter	N0	43 (48%)
	N1	31 (35%)
	N2	15 (17%)
M parameter	M0	45 (52%)
	M1	43 (48%)
Vascular invasion		16 (18%)

*N* number of patients

## Methods

### Tissue microarray construction

Formalin-fixed paraffin-embedded tissue blocks with representative tumor areas with at least 80% of tumor cells were identified through a review of corresponding hematoxylin–eosin (HE) stained slides. Areas of interest were identified and marked on each selected block. The tissue microarrays paraffin block was cored using a 2-mm core. For each case, two or three representative cores of tumor were arrayed. The cores were transferred to the “donor block” using an automated tissue arrayer (TMA Master; 3DHISTECH, Budapest, Hungary). Next, paraffin-embedded TMA block was cut into 3–4 μm-thick sections, using a manual rotary microtome (Accu-Cut, Sakura Finetek, Torrance, CA, USA). The prepared sections were then placed on extra adhesive slides (Superfrost Plus; Menzel-Glaser, Braunschweig, Germany).

Immunohistochemical staining was performed using DakoAutostainer Link 48 (Dako, Agilent Technologies, USA) and BenchMark® Ultra automated slide processing system (Ventana Medical Systems, Tucson, AZ, USA). The following primary antibodies were used: rabbit monoclonal anti-CD44 (SP37) antibody (ready to use, Ventana Medical Systems, Tucson, AZ, USA), rabbit polyclonal anti-DCLK1 antibody (HPA015655, Sigma-Aldrich, Merck KGaA, Darmstadt, Germany), rabbit polyclonal anti-LGR5 antibody (HPA012530, Sigma-Aldrich, Merck KGaA, Darmstadt, Germany), and rabbit polyclonal anti-ANXA2 antibody (HPA046964, Sigma-Aldrich, Merck KGaA, Darmstadt, Germany). In the beginning, standardization and optimization of the IHC method were performed on a tissue recommended based on the antibody datasheet and reference sources (The Human Protein Atlas: https://www.proteinatlas.org) (Uhlen et al. 2010).

### Immunohistochemical staining of LGR5, ANXA2, and DCLK1

Immunohistochemical staining of anti-LGR5, anti-ANXA2, and anti-DCLK1 antibodies was done using the DakoAutostainer Link 48 and Dako PT Link pre-treatment module. Prepared slides with tissue sections were deparaffinized and rehydrated. In the first step, the slides were heated in a high-pH buffer (Dako, Agilent Technologies, USA) at 95–98 °C for 20 min in PT Link (Dako, USA) for antigen retrieval. Then, the endogenous peroxidase activity was inhibited using 3% H2O2 for 10 min at room temperature (RT). The slides were treated with 3% bovine serum albumin solution for 15 min at RT to block non-specific antibody binding sites. Next, the sections were incubated with rabbit polyclonal anti-DCLK1 antibody (1:100), rabbit polyclonal anti-LGR5 antibody (1:500), and rabbit polyclonal anti-ANXA2 antibody (1:100) for 30 min. Subsequently, slides washed three times with phosphate-buffered saline. After adding the secondary horseradish peroxidase (HRP, Dako, Agilent Technologies) labeled antibody for 20 min at RT, the 3,3′diaminobenzidine (DAB) was used to detect the localization of the antigen–antibody complex. The sections were counterstained in hematoxylin and washed. Finally, tissue sections were dehydrated in increasing ethanol concentrations (80, 90, 96, and 99.8%), cleared in xylenes (I–IV), mounted using mounting medium, and observed.

### Immunohistochemical staining of CD44

Immunohistochemical staining of anti-CD44 was done using the BenchMark® Ultra automated slide processing system (Ventana Medical Systems, Tucson, AZ, USA). In the first step, deparaffinization and rehydration were performed in EZ Prep solution (Ventana Medical Systems, Tucson, AZ, USA). Next, antigen retrieval of tissue sections was performed in Cell Conditioning (CC2) solution for 68 min. Incubation with the primary rabbit monoclonal anti-CD44 (SP37) antibody was performed for 20 min at 36 °C. The reaction was performed using the visualization system (UltraView DAB Detection Kit; Ventana Medical Systems, Tucson, AZ, USA). The slides were counterstained with Hematoxylin II for 12 min and Bluing Reagent for 4 min. Finally, tissue sections were dehydrated in increasing ethanol concentrations (80, 90, 96, and 99.8%), cleared in xylenes (I–IV), mounted using mounting medium, and observed.

### Evaluation of immunohistochemistry staining

The pathologists who were evaluating the immunohistochemical expression of examined antigens worked independently, and they were blinded from the patients’ clinical, as well as other data. The protein expression was evaluated using light microscope ECLIPSE E800 (Nikon Instruments Europe, Amsterdam, Netherlands) at 20× original objective magnification. The staining intensity was measured on a four-point scale as negative (0), week (1), moderated (2), and strong (3). The percentage of IHC positive cells were also recorded. The H-score was assigned due to calculation of the percentage of cells at each staining intensity level according to the following formula: H-score = [1 × (% cells 1 +) + 2 × (% cells 2 +) + 3 × (% cells 3 +)]. The H-score amounted maximal 300 and minimal 0 points (Yeo et al. 2015).

### Statistical analysis

All statistical analyses were performed using Statistica version 13 (StatSoft) and Microsoft Excel 2007. The expression values of analyzed proteins were presented 25th percentile (25p), the median (M), and the 75th percentile (75p). The comparative studies were analyzed statistically using the nonparametric *U* Mann–Whitney and Kruskal–Wallis test. Overall survival (OS) curves were designed using ROC curves and performed with the Kaplan–Meier method and compared with the log-rank test*.* The *p* value < 0.05 was considered statistically significant.

## Results

**Fig. 1:**
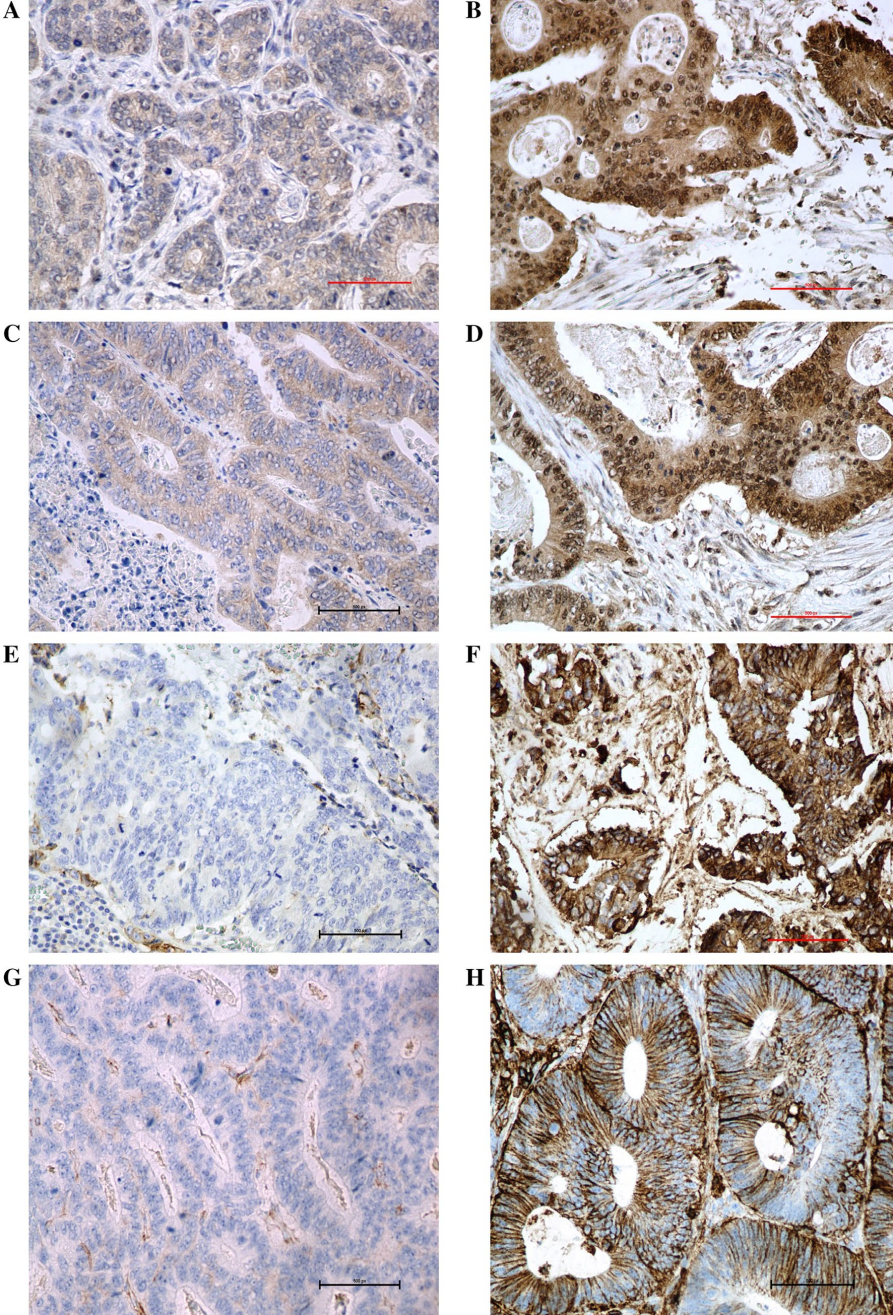
Representative images of cancer stem cell markers by immunohistochemistry in colorectal cancer tissue with **a** low expression of Lgr5, **b** high expression of Lgr5, **c** low expression of DCLK1, **d** high expression of DCLK1, **e** low expression of ANAX2, **f** high expression of ANAX2, **g** low expression of CD44, and **h** high expression of CD44

In the current study, there was observed the nucleocytoplasmic expression of Lgr5, DCLK1, and ANXA2 (Fig. 1) and membranous expression of CD44 had. The expression of Lgr5 was found in 92% of samples, DCLK1 in 97% cases, ANXA2 in 88% cases, and CD44 in 82% cases (Tables 2, 3). In most of the cases, a co-expression of all investigated CSC markers was detected (Table 4). Additionally, the presence of stromal markers LGR5, CD44, ANXA2, and DCLK1 staining has been identified as a common finding among adjacent microenvironment of the CRC.

**Table 2:** The expression of stem cell markers (CSC) in investigated tissue

CSC marker	The presence of proteins expression	*N* (%)
Lrg5	Positive (+)	82 (92%)
	Negative (–)	7 (8%)
DCLK1	Positive (+)	87 (97%)
	Negative (–)	2 (3%)
ANXA2	Positive (+)	79 (88%)
	Negative (–)	10 (12%)
CD44	Positive (+)	73 (82%)
	Negative (–)	16 (18%)

*N* number of cases

**Table 3:** Results of IHC examination according to H-score system

Results in H-score	CD44 [%]	ANXA2 [%]	DCLK1 [%]	Lgr5 [%]
< 30	41.6	42.7	9.0	13.5
30–100	30.3	41.6	48.3	42.7
100–200	22.5	15.7	42.7	32.6
200–300	5.6	0.0	0.0	11.2

**Table 4:** Different population of detected cancer stem cells in colorectal cancer

Co-expressed CSC markers	Number of cases	Percentage value [%]
CD44(+)ANXA2(+)DCLK1(+)Lgr5(+)	64	72
CD44(–)ANXA2(+)DCLK1(+)Lgr5(+)	12	13
CD44(+)ANXA2(–)DCLK1(+)Lgr5(+)	5	6
CD44(+)ANXA2(+)DCLK1(+)Lgr5(–)	3	4
CD44(+)ANXA2(–)DCLK1(+)Lgr5(–)	2	2
CD44(–)ANXA2(–)DCLK1(+)Lgr5(+)	1	1
CD44(–)ANXA2(–)DCLK1(+)Lgr5(–)	1	1
CD44(–)ANXA2(–)DCLK1(–)Lgr5(–)	1	1
	*n* = 89	100%

**Fig. 2:**
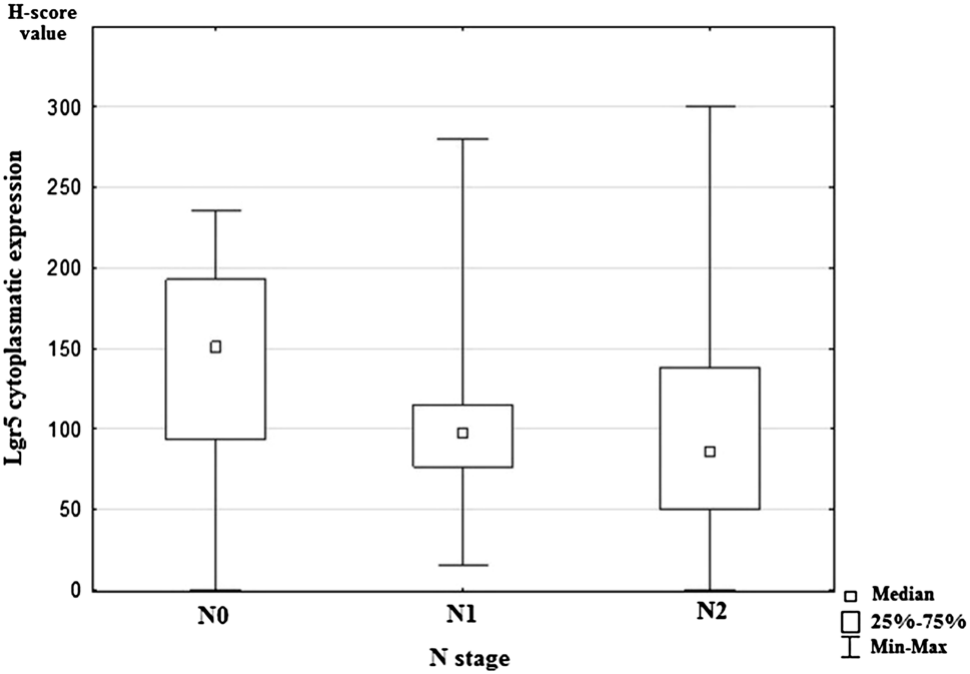
Median nuclear expression of Lgr5 for the studied groups according to regional lymph-node status. N0: colorectal cancers without nodal metastases, N1: colorectal cancers with 1–3 positive regional lymph nodes; N2: colorectal cancers with four or more positive regional lymph nodes

**Fig. 3:**
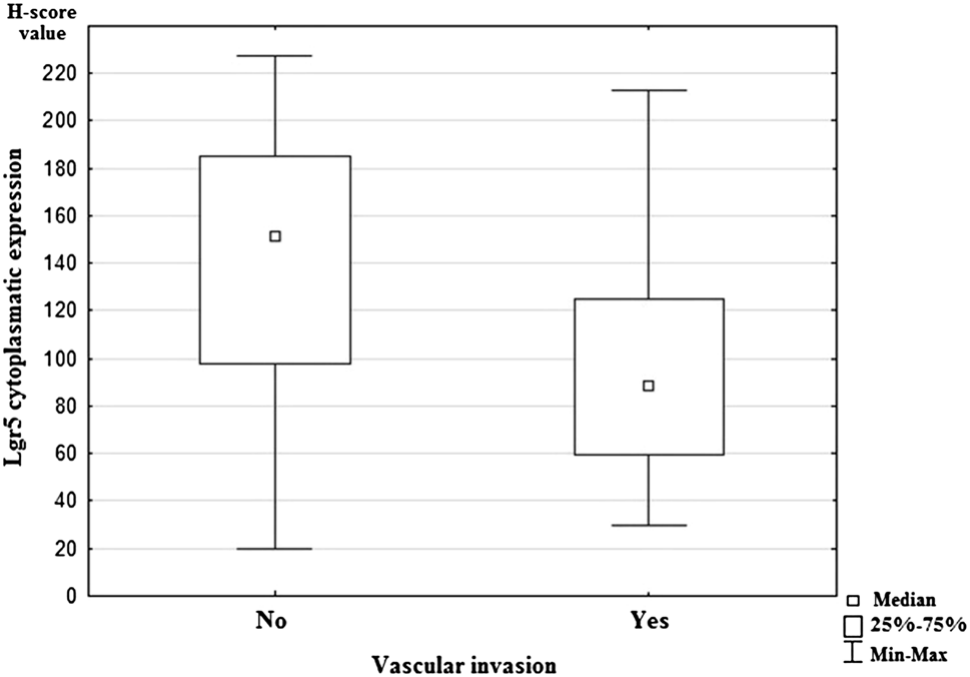
Median nuclear expression of Lgr5 for the studied groups according to vascular invasion

Statistical analysis showed significant differences in expression of Lgr5 and the presence of lymph-node metastases (*p* = 0.011). The study revealed a lower expression of Lgr5 in both pN1 and pN2 status compared to pN0 (Fig. 2). Moreover, higher Lgr5 expression was statistically significantly related to vascular invasion in examined cancer tissue samples (*p* = 0.027; Fig. 3). However, statistical analysis did not show any correlations between Lgr5 and tumor localization, grade, T stage, the presence of distant metastases, and infiltration of neural structures. The remaining investigated CSC markers (DCLK1, ANXA2, and CD44) did not demonstrate a statistically significant relationship with tested clinicopathological features of colorectal cancer. All results are shown in Table 5.

**Table 5:** The correlations between expression of Lgr5, DCLK1, ANXA2, and CD44 and clinicopathological features of colorectal cancer

		Lgr5				DCLK1				ANXA2				CD44			
		25p	M	75p	*p*	25p	M	75p	*p*	25p	M	75p	*p*	25p	M	75p	*p*
G	W/M	80.63	100	173.8	ns	81	100	110	ns	16.88	38.75	75	ns				
	Poor	55	87.5	158.8		72.5	115	135		7.5	96.25	102.5		3.75	45	75.63	ns
T	pT2	63.75	95	162.5	ns	82.08	96.67	121.7	ns	3.33	27.50	51.25	ns				
	pT3	76.67	100	172.5		81.00	100	110.8		13.75	42.08	79.50		8.75	66.67	128.1	
	pT4	62.50	95.00	158.8		75.83	100	121.7		20.00	41.25	118.3		6.25	25	96.25	
N	pN0	90	151.3	194.5	0.011	79.38	96.67	115.8	ns	27.63	48.75	75	ns	2.92	65	156	ns
	pN1	70.83	97.5	117.5		88.33	100	110		5	31.25	74.17		5	63.33	117.5	
	pN2	47.5	86.25	151.8		75	92.5	105		6.87	25.83	92.75		5	58	71.67	
M	pM0	63.75	110	175	ns	80	96.67	115	ns	18.33	40	68.33	ns	5	65	127.5	ns
	pM1	78.33	100	150		76.98	100	110		7.5	37.5	102.5		7.5	56.67	105	
Vas	v1	57.19	88.75	137.5	0.027	76.88	102.9	132.5	ns	12.50	38.75	78.96	ns	8.12	66.46	131.3	ns
	v0	96.25	151.3	185		76.04	93.33	107.5		20	45.83	73.75		5.42	102.5	175.8	
Nif	ni1	168.8	185	211.7	ns	78.75	90.00	103.3	ns	12.5	50.00	75.00	ns	0	3.34	5	ns
	ni0	84.58	150	180		71.88	92.92	110		11.25	29.17	61.04		20.63	88.75	173.8	

*G* grade, *W/M* well/moderated, *T* the size of the primary tumor/deep of primary tumor invasion, *pT2* the invasion of muscularis propria only, *pT3* the invasion of subserosa or surrounding tissues, *pT4* the presence on visceral peritoneum or attached to neighboring structures, *N* metastases to regional lymph nodes, *pN0* lack of regional lymph-node metastases, *pN1* the tumor cells found in 1–3 regional lymph nodes, *pN2* the tumor cells found in more than four regional lymph nodes, *M* distal metastases. *pM0* lack of distal metastases, *pM1* presence of distal metastases, *Vas* vascular invasion, *v1* angioinvasion in investigated tissue, *v0* lack of angioinvasion in investigated tissue, *Nif* neural infiltration, *ni1* neural infiltration in investigated tissue, *ni0* lack of neural infiltration in investigated tissue

**Fig. 4:**
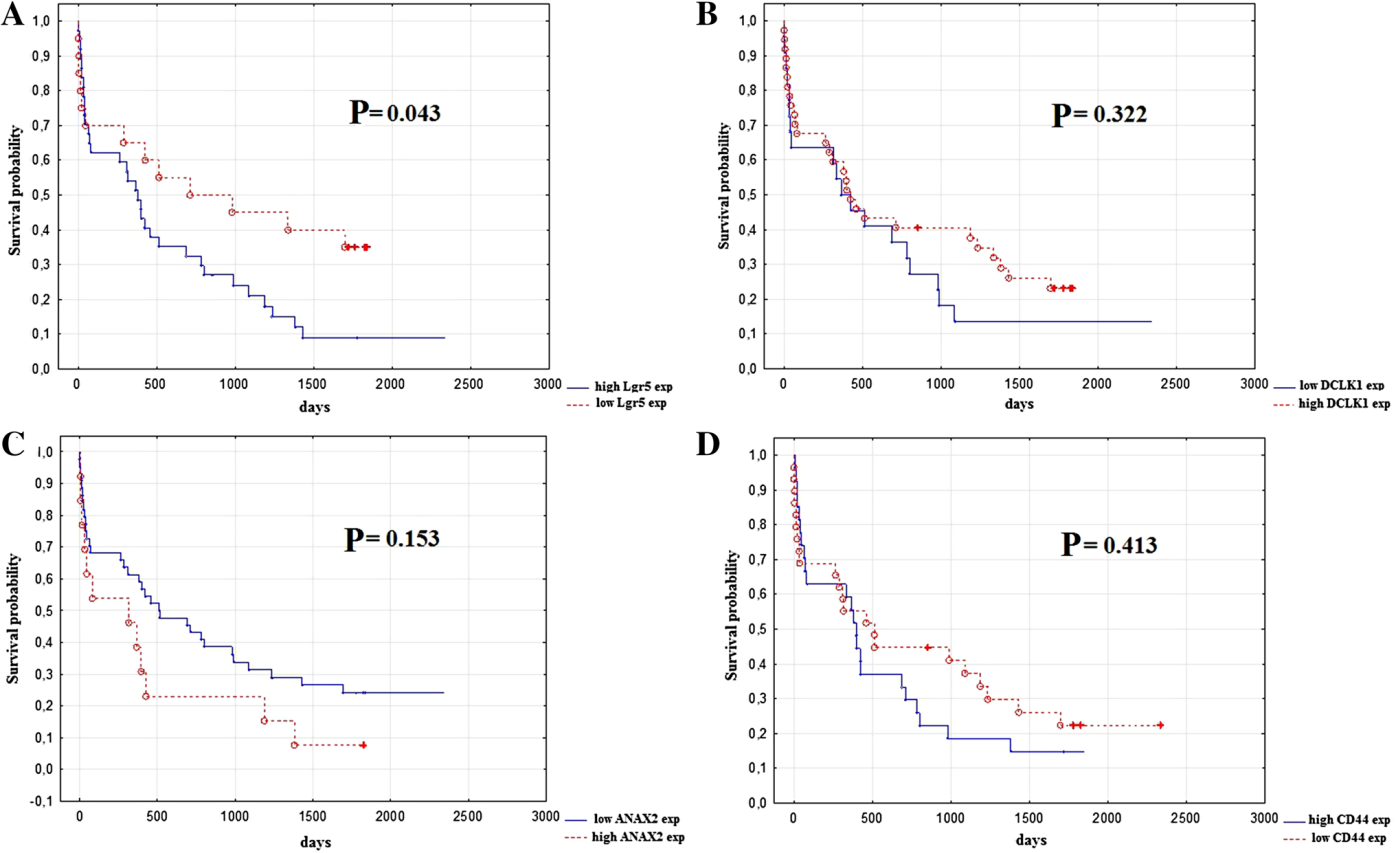
Stem cell marker expressions in primary tumor and overall survival in case of **a** Lgr5; **b** DCLK1; **c** ANXA2; and **d** CD44

The ROC curves were performed to receive the optimal cut-off points for the expression level of all investigated CSC markers. The expressions levels of Lgr5, DCLK1, ANXA2, and CD44 were divided into two groups, the first with a high expression and the second with a low expression. The obtained results were analyzed in correlation with an OS after a primary surgery by use of Kaplan–Meier survival curves (Fig. 4). In the case of Lgr5, a high *H*-score value was significantly related to reduced OS rate (*p* = 0.043). The remaining proteins did not demonstrate a statistically significant correlation.

## Discussion

The current study aimed to investigate the expression of commonly occurring cancer stem cell markers of colorectal cancer. Our study showed that increased Lgr5 expression is statistically significantly related to a lack of lymph-node metastasis, presence of vascular invasion, and low survival rate in CRC. Moreover, the determination of CSC expression in postoperative tissue could be used in clinical practice as a prognostic factor but also to help with treatment planning.

Lgr5+ cells have been frequently investigated as CSC in colorectal cancer (Shimokawa et al. 2017). The expression of Lgr5 occurs between 56.3% and 82.4% in comparison to between 6 and 25% of Lgr5+ cells among crypt cells of the normal intestine (Ziskin et al. 2013; He et al. 2014; Jia et al. 2015; Zheng et al. 2018; Wang et al. 2018b). Furthermore, the series of studies have suggested that enhancement of Lgr5 expression is correlated with an evolution from adenoma to adenocarcinoma. (Fan et al. 2010; Zheng et al. 2018). In our study, 92% of exanimated colorectal specimens were Lgr5+ . The overexpression of Lgr5 in the colorectal cancer cells occurs due to up or downregulation of several noncoding RNAs, such as CASC15 miR-4310 or miR-23a, as a consequence of activation of PI3K/Akt signaling pathway (Takahashi et al. 2011; Mukohyama et al. 2017). Other studies have also emphasized the role of EGF/EGFR/STAT3/PDGFA pathway and histone acetylation on the Lgr5 promoter (Cheng et al. 2017, 2018).

From the previous studies on CRC, it is known that Lgr5 enhances downstream Wnt/β-catenin signaling and Lgr5+ cells exhibit a high ability of colony formation, self-renewal, differentiation, and Ki67 proliferative index (Fan et al. 2010; Takahashi et al. 2011; He et al. 2014; Leng et al. 2018; Zheng et al. 2018; Salehizadeh et al. 2019). The recent works have emphasized that a high level of this CSC marker is positively correlated with regards to both tumor size and the depth of invasion into surrounding tissues. (Fan et al. 2010; Hsu et al. 2013; Jiang et al. 2015; Nishioka et al. 2018; Zheng et al. 2018). Zhou et al. observed in their study that patients with CRC had reduced Lgr5 expression in more advanced stages (Zhou et al. 2017). However, our result did not show any correlation between Lgr5 and primary tumor size (T stage), which is supported by He et al.’s study, with a similar conclusion to our finding (He et al. 2014). A complete explanation of this issue needs more research; nevertheless, our results suggest a different role of Lgr5 in cancer development.

Moreover, some studies suggest that the degree of cancer differentiation (grading) is also highly associated with Lgr5 expression (Wu et al. 2012; Wang et al. 2018b). However, a couple of studies have demonstrated a low level of Lgr5+ cells in high grade, poorly differentiated CRC (Dame et al. 2018; Sato et al. 2019). In our study, a statistically significant correlation between histological grade and Lgr5 expression was not observed. Similar lack of relationship was also observed by other authors in their studies (He et al. 2014; Jiang et al. 2015).

**Fig. 5:**
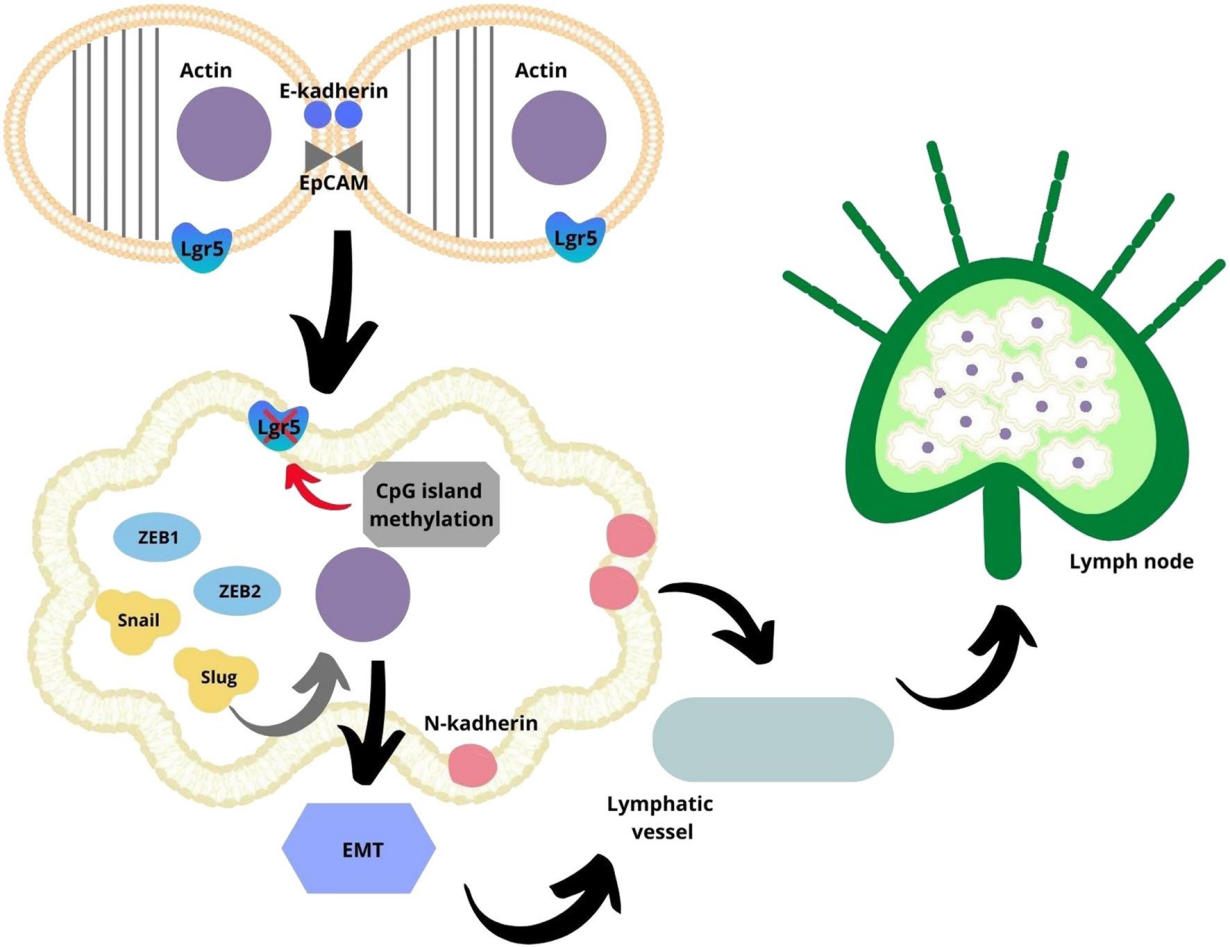
The cell adhesion is increased due to enhance levels of cortical F-actin in colorectal cancer cells with overexpression of Lgr5. The colorectal cancer cells could change their phenotype into mesenchymal-like with high expression of Snail, Slug, Zeb1 and 2, and N-cadherin and low expression of epithelial-associated genes such as E-cadherin after the downregulation of Lgr5 due to progressive CpG island methylation, what lead to epithelial–mesenchymal transition and induction of metastatic process (Walker et al. 2011; Carmon et al. 2017; Jang et al. 2018; Leng et al. 2018). EpCAM—epithelial cell adhesion molecule; Lgr5—leucine-rich repeat-containing G-protein-coupled receptor 5; ZEB1—zinc-finger E-box-binding homeobox 1; ZEB2—zinc-finger E-box-binding homeobox 2; Snail—zinc-finger protein SNAI; Snug—zinc-finger protein SNAI2; EMT—epithelial–mesenchymal transition

The association Lgr5 and metastatic ability of CRC seem to be even more controversial than the above-discussed parameters. Our results suggest that the expression of Lgr5 is significantly lower in CRC with lymph-node metastases. However, the majority of until now published studies reported the opposite relationship between these features in CRC (Wu et al. 2012; He et al. 2014; Jiang et al. 2015; Zheng et al. 2018; Wang et al. 2018b). Most of the authors explained that the main roles of CSC are to migrate to the target tissue and form secondary tumors. The recent advancement in molecular biology seems to give the indications to understanding the underlying molecular pathways, which could make our result more reliable. Studies have shown that overexpression of Lgr5 increases cells adhesion due to enhance levels of cortical F-actin (Carmon et al. 2017; Walker et al. 2011). On the other hand, Lgr5- subpopulation of CRC CSC seems to be characterized by a higher expression of mesenchymal-associated genes such as Snail, Slug, Zeb1 and 2, and N-cadherin, and by a lower expression of epithelial-associated genes such as E-cadherin, occluding, and epithelial cell adhesion molecule (EpCAM) (Leng et al. 2018). According to some studies, these events could be explained by the fact that the sphere formation of CSC (and possible EMT) is associated with a decreased Lgr5 expression due to progressive CpG island methylation of its promoter during the progression of tumorigenesis (Jang et al. 2018). To summarize, due to downregulation of Lgr5 expression, intracellular mechanisms promoting cell adhesion may decrease with EMT promotion, leading to an increased metastatic ability of CSC. This mechanism seems to explain why a low expression of Lgr5 in a primary tumor may correlate with the presence of lymph-node metastases observed in the current study (Fig. 5). However, our results did not confirm any correlation between Lgr5 expression and distal metastasis.

Our analysis of Lgr5 expression and OS showed that a high level of Lgr5 could decrease the patient’s overall survival time. This remark suggests that Lgr5 could be used in clinical practice as a potential prognostic factor. A similar conclusion of a high level of Lgr5 is an unfavorable factor with a shorter OS and disease-free survival (independent from tumor size) was also given in many other studies (Takahashi et al. 2011; Wu et al. 2012, 2016; Hsu et al. 2013; Jiang et al. 2015; Nishioka et al. 2018). There are a couple of possibilities that could explain this observation. First, our study revealed the high expression of Lgr5 to be correlated with vascular invasion, which may indirectly indicate the presence of some unknown mechanism that may contribute to the ability of cancer cells to invade the vessels. It has been shown that Lgr5 enhances downstream Wnt/β-catenin signaling and consequently deregulation of factors such as c-MYC or CDKN1A. (Fan et al. 2010; Takahashi et al. 2011; He et al. 2014; Zheng et al. 2018). WNT signaling cascades in Lgr5+ cell cross-talk with other pro-tumorigenic components, for example, the FGF, Notch, Hedgehog, and TGFβ/BMP signaling, and promotes EMT of Lgr5+ cells (Wu et al. 2016; Jang et al. 2018; Solomon et al. 2018; Zhang et al. 2018). Second, CSCs are a subpopulation of cancer cells that are resistant to chemotherapy. Several studies suggested Lgr5+ as resistant not only to 5-FU but also to other components of commonly used CRC chemotherapy regimens such as Oxaliplatin and Irinotecan (Kobayashi et al. 2012; Zhang et al. 2019). Balancing both observations, it might be possible to deduce that high subpopulation of Lgr5+ cells due to increased spread of cancer cells including CSC into bloodstream creates a CRC cellular pool, which survives resection of the primary tumor, and is then responsible for resistance to adjuvant chemotherapy. Furthermore, conversion of LGR5+ to LGR5- CSC has been shown to enhance drug resistance in CRC (Hou et al. 2018). It could be possible that the subpopulation of Lgr5+ CSC grow during cancer progression and represent CRC cells, which could change into chemo-resistant Lgr5- cells with metastatic ability during cancer progression. This suggestion could explain why a weak expression of Lgr5 in CRC correlate with lymph-node metastasis (high population of present Lgr5- CSC at the time of diagnosis) and its high level with worse overall survival (the detection of all cells, which might transform into metastatic-related chemo-insensible clone). However, Wang et al. suggested the only nonsignificant relationship of Lgr5 alone with OS and Lgr5 has been in addition described in Jiang et al.’s study, different from our results as prognostic marker for better clinical outcome in CRC patients (Wang et al. 2018a; Zheng et al. 2018).

Our study did not show any significant correlation between CD44, DCLK1, and ANXA2 expression and clinicopathological features of colorectal cancer and OS. Recent studies have also demonstrated no significant association between CD44 and tumoral characteristics of CRC (Rohani et al. 2017; Wang et al. 2019). However, some studies have shown that CD44 overexpression has been correlated with worse OS of patients with CRC (Wang et al. 2019,2017). Similarly, several studies show a positive correlation between high DCLK1 expression and CRC clinicopathological characteristics and poor OS (GZIL et al. 2019a, b). However, Dai T. et al.’s study reveals that a high DCLK1 expression in CRC tissue could play a protective role against tumor progression and correlates with longer survival time (Dai et al. 2018). Furthermore, recent studies suggested a statistically significant correlation between high ANXA2 protein expression and histological grade, pTNM stage, and worse OS than in patients with low ANXA2 (Duncan et al. 2008; Yang et al. 2013).

Although it was not the aim of our study, we observed the expression of all investigated CSC markers in the stroma of CRC samples. Previous studies are consistent with our findings. An earlier study suggested that stromal staining of LGR5 could be associated with cancer with an advanced stage of CRC (Dame et al. 2018). Others have identified the presence of Lgr5+ stromal cells in the oral mucosa and in lung alveolar mesenchyme (Boddupally et al. 2016; Lee et al. 2017). In case of CD44, this protein is a cell surface glycoprotein expressed commonly on lymphocytes, monocytes, and granulocytes and it has been interestingly suggested, that the absence of stromal CD44 expression was connected with an increased death rate of patients with CRC (Furuta et al. 1996, 1998; Cairns et al. 2001). Latest studies highlighted the role of ANXA2 expression in cancer-associated fibroblasts in stromal tissue in pancreatic ductal carcinoma and epithelial ovarian cancer as a predictive biomarker for overall survival (Paliwal et al. 2012; Christensen et al. 2019). Other studies indicate that DCLK1 has been shown to be expressed in the stroma of colon, pancreatic, prostate, breast cancers, esophageal adenocarcinoma, and non-small cell lung cancer (NSCLC) (Sureban et al. 2011; Whorton et al. 2015; Tao et al. 2017). Moreover, Tao A. et al.’s study reveals association between DCLK1 expression in NSCLC cells and the tumor stroma and their correlation with worse prognosis (Tao et al. 2017). Future studies should focus on complex understanding of the significance of CSC markers-expressing stromal cells in colorectal carcinogenesis.

In conclusion, our results suggested a strong relationship between CSC marker Lgr5 and vascular invasion, presence of lymph-node metastasis, and poor survival. Our study suggests Lgr5 as an unfavorable prognostic factor and its high level in CRC could direct to a more precise classification of treatment and observation in patients with a shorter survival life-span. Dualistic nature of Lgr5+ CSC, of which decreased expression is correlated with nodal spread and increased with vascular invasion and shorter overall survival, needs to be more investigated, because our results in combination with earlier molecular biology studies suggest interesting nature of those cells.

## Data Availability

Author decelerates that all data and material are available by corresponding author.
